# The effect of digitalization of nursing forms in ICUs on time and cost

**DOI:** 10.1186/s12912-023-01333-6

**Published:** 2023-06-13

**Authors:** Nevin Yilmaztürk, İlker Kose, Sinem Cece

**Affiliations:** 1 Department of Health Management, Istanbul Medipol University, Istanbul, 34810 Turkey; 2 Department of Computer Engineering, Alanya University, Antalya, 07400 Turkey; 3 Alanya University, Antalya, 07400 Turkey

**Keywords:** Medical informatics, Nursing informatics, Digital technology, Time management, Health care costs

## Abstract

**Objective:**

Intensive Care Units are one of the areas with the lowest digitization rate. This study aims to measure the effect of digitizing medical records kept in paper forms in ICUs on time-saving and paper consumption. In our study, care forms in ICUs were transferred to digital media. In our research, care forms in ICUs were transferred to digital media.

**Methods:**

The time required to fill out the nursing care forms on paper and digital media was measured, the change in paper and printer costs was determined, and the results were compared. Two volunteer nurses working in the ICU of a university hospital in Istanbul measured the time it took to fill out the forms of patients on paper. Then, a future projection was made using digital form data of 5,420 care days of 428 patients hospitalized between October 2017 and September 2018. Only anonymous data of patients hospitalized in the general ICU were used, and other untempered were not included in the study.

**Results:**

When the forms were filled in digitally by the nurses, one nurse per patient per day saved 56.82 min (3.95% per day).

**Discussion:**

Health care services are provided in hospitals in Turkey with 28,353 adult intensive care beds and an occupancy rate of 68%. Based on the occupancy rate of 68%, the number of full beds is 19,280. When 56.82 min are saved per bed from the forms filled by the nurses, 760.71 care days are dedicated. Considering the salary of 1,428.67 US dollars per nurse, the savings to be achieved are estimated to be 13,040,804.8 US dollars per year.

## Objective

### Digitalization in nursing services

The digitalization of health care delivery has brought up a new relationship for nurses; the nurse and technology relationship. Although technology cannot replace compassionate inpatient care, it is defined as a unique competence expected to be acquired in nursing [1]. This competence, believed to be developed through informatics expertise and health informatics solutions, is a significant opportunity to improve health care quality [2].

In addition to keeping the patient’s information wholly and accurately, health informatics has made it possible to access this information easily and quickly. This ease of access positively impacts correct decision-making, reduces medical errors, and supports evidence-based practices. It is also known that health information systems that provide easy access to nursing care plans enhance satisfaction in nurses. For example, it is determined that the facilitation of information transfer in nursing shifts, resulting from information systems, increased the satisfaction rate among nurses by 34% [3]. There are also findings that health information systems reduce nurses’ workload and improve the quality of nursing care [4].

It seems that the time nurses spend on documentation is longer than the time allocated directly to patient care [5]. In addition, errors due to physical and cognitive fatigue of the person are likely to occur regarding the forms that are manually filled out. A study conducted in a public hospital in Turkey in 2013 established that nurses fill out approximately 68 registration forms, other than those for daily nursing care services, in addition to the forms used in all branches. Thirty of these forms (44.2%) are directly related to the practice of the nursing profession. Apart from these, 38 (55.8%) forms are patient registration forms as well as forms and reports related to devices and technical services, which are under the responsibility of physicians or other unit managers (professionals other than nurses) [6].

Patients treated in ICUs (ICUs) are at higher risk of being exposed to medical errors and patient safety issues than patients in other branches [7]. Fortunately, advancing technology offers modern healthcare providers innovative ways to solve everyday challenges in improving patient care and processes. Studies show that the use of electronic health records in the ICU is increasing. Studies show that the use of electronic health records in the ICU is rising. The resulting electronic health record is integrated with clinical decision support systems, contributing to the correct treatment of the right patient [8].

A study conducted at Akdeniz University Hospital ICU in 2017 determined that nurses use technology effectively in patient care and adopt a positive attitude towards technology [9]. Another study found that adopting technology in intensive care contributed to developing nursing care and facilitated nursing care processes. Thus, it was observed that nurses could spend more time helping patients with their emotions, such as anxiety and worry [10].

When the specialized areas of ICUs (palliative care, post-surgery, etc.) were examined, it was observed that the quality of nursing care was directly associated with mortality rates, costs, complications, and infection. 797 patients admitted to the ICU of Georgia Atlanta Urban Training Hospital between July 2010 and June 2011 were examined before and after electronic health records. The study revealed that computer-assisted physician order entry (CPOE) in the intensive care environment diminished medication errors and reduced mortality rates [11].

Another study monitoring the patient population change in the surgical ICU over time revealed that the catheter-related infection rate was 85%, and the mortality rate was 28%. Additionally, the pre-diagnosis rate in the first 48 h increased from 17.8 to 20.8. These results showed that electronic health records play an essential role in the quality of care. It has been determined that nurses in the ICU allocate 32% of their work activities to indirect care, namely documentation [12].

In a study conducted in 2011 with a research group consisting of 9 clinical nurses, 27 nurse technicians, 24 nurse assistants, and a nurse coordinator in the ICU of a private hospital in the Brazilian state of Ribeirão Preto, the average nursing care time was found to be 29.5 h per patient/day. And it was determined that 27.4 h of this maintenance consisted of direct supervision and 2.1 h of indirect maintenance [13]. The use of digital order has also reduced the time spent by ICUs for documentation. Thus, the time spent by nurses for patient care has increased [14].

In another study conducted in Australia in 2017, while all these critical situations were experienced, it was determined that there was an 85% decrease in the error rates in drug prescribing, with the use of computerized physician order entry instead of paper-based medication orders in ICUs. More importantly, it was determined that there was a 12% decrease in ICU mortality rates thanks to the computerized physician order application [15].

A study published in 2020 showed that the rate of ventilation-associated pneumonia was reduced from 4.5 to 0.5% by revising the nursing care forms in the intensive care service of Izmir Tire State Hospital and integrating them with the clinical decision support system [16].

## Materials and methods

Within the scope of the digital transformation project in hospitals initiated by the Ministry of Health in Turkey in 2013, the use and adoption of technology in hospitals are regularly measured [17]. The Electronic Medical Record Adoption Model (EMRAM) model developed by (Healthcare Information and Management Systems Society) HIMSS is used for this measurement [18]. EMRAM consists of 8 levels, ranging from 0 to 7. Bahçelievler State Hospital is validated as Stage 6 based on the EMRAM criteria, thanks to the digital hospital studies conducted by this hospital. One of these studies is the digitalization of paper forms in ICUs.

The research question of this study is, “Does digitalization provide time and cost benefits in intensive care units?“. “Does it benefit the working conditions of nurses?“

### Number of patients and care times

Our study is based on the daily care data of 5,420 days for 428 patients treated in the General ICU at Bahçelievler State Hospital between October 2017 and September 2018. The data was acquired by exporting them in an MS Excel (Microsoft Inc., Redmond, WA, ABD) list after being anonymized from the MSSQL (Microsoft Inc., Redmond, WA, ABD) database through the Bahçelievler State Hospital Information Management System (SARUS HIS, Istanbul, TR) used by the hospital. Different results can be expected with different HISs. However, in any case, it can be argued that digitalization in ICU saves time and paper. We aim to reveal the financial value of the envisaged savings.

### Analyzed ICU forms

The forms, which were filled out on a routine basis for all patients at the specified stages in the Intensive Care workflow and repeated at certain times, were determined through a descriptive study conducted with intensive care nurses. The forms used in exceptional cases (only for some special cases) are excluded. The forms used in the study are:

1.Planning and Care Forms.

- Patient Evaluation Form.
- Diabetes Patient Follow-Up Form.
- Pressure Sore Risk Assessment Form (Pressure Sore Risk Assessment Form).
- Nutrition Risk Assessment Form.

2. Daily Follow-Up Form.

All the forms mentioned above will be referred to as “daily forms.”

### Measuring form filling time in paper and digital media

The displays featured in the XXX (SARUS HIS, Istanbul, TR) application developed explicitly by SARUS HIS for intensive care are designed to cover all paper forms used in this study.

The filling time for clinical forms in paper and the digital environment was measured by the observational research method. Considering the voluntary basis, two intensive care nurses were selected, one with experience of 8 years (Nurse X) and the other with experience of 4 years (Nurse Y).

The time required to fill out paper forms was recorded by measuring the time needed by nurses to fill out the relevant forms at the bedside. Digital form filling time was measured using XXX application interfaces. In both measurements, two nurses were asked to fill out each type of form at least 3 times on paper and digital media at different times, and the time was measured. The duration spent filling out forms on paper and digital media was measured six times for each form. The following formula was used to calculate the mean difference between both periods.

Where;

F_i_: Nursing forms used in ICU.

n: Number of nursing forms.

N_j_: Nurse.

k: Number of nurses.

p: Paper-based form filling time.

d: Digital form filling time.

Then, the time difference between all nursing forms in the figures was calculated as follows:

$$Time\, difference=\frac{\sum _{i=1}^{n}\sum _{j=1}^{k}{(p}_{ij}-{d}_{ij})}{6}$$

### Determination of paper consumption

The calculation for the consumption of paper forms is as follows.

Where;

*P: number of pages of the paper-based nursing clinical form*

*R: number of repetitions of the paper-based nursing clinical form in a day*

*S: length of stay (days)*

*I: paper-based nursing clinical form type*

*N: number of inpatients*

Then, the number of paper-based clinical nursing forms was calculated as follows:

$$number of pages= \sum _{p=1}^{n}\sum _{i=1}^{3}{P}_{i}{R}_{i}{S}_{p}$$

### Calculating savings on a national scale

*C: monthly gross cost of nurses*

*K: number of nurses (in Turkey)*

$$Nurse\, Cost\, Savings=\frac{Time\, difference}{24\times 60}x\sum _{i=1}^{K}{C}_{i}$$

The most commonly used form in ICU is a daily follow-up form consisting of 11 parts, and it is filled out every hour 24 times a day. Within the scope of digitalization, 7 of these 11 sections were automatically transferred from medical devices to XXX. The data in the remaining 4 fields were recorded in the manual XXX by nurses. Therefore, the time for filling out these fields was deemed 0 seconds.

This study has the approval of Istanbul Medipol University “Non-interventional Scientific Research” ethics committee.

## Results

### Effect of digitalization on the time spent by nurses to fill out forms

The time for filling out clinical nursing forms in paper and digital environments is shown in Table 1.

**Table 1:** Nursing forms and filling times in the ICU

Frequency of Recurrence (Day)	Form Name	Digital Form (sec)	Paper Form (sec)	Digital Form (sec)	Paper Form (sec)	Digital Form (sec)	Paper Form (sec)	Digital Form (sec)	Paper Form (sec)	Digital Total Time (sec)	Paper Total Time (sec)	Time Difference (sec)	Saving Rate
		X Nurse		Y Nurse		X Nurse		Y Nurse		Average		Average	
		For once				One Day							
	**Planning and Care Form**												
0,033	Patient Evaluation Form	600	600	540	780	20	20	18	26	19	23	4	17,39%
2	Diabetes Patient Follow-Up Form	15	60	15	120	30	120	30	240	30	180	150	83,33%
2	Pressure Sore Risk Assessment Form	20	60	25	120	40	120	50	240	45	180	135	75,00%
2	Nutrition Risk Assessment Form	25	180	35	300	50	360	70	600	60	480	420	87,50%
24	**Daily Follow-Up Form**												
	Vital	0	240	0	300	3.360	5.760	4.200	7.200	3.780	6.480	2.700	41,67%
	Respiration	0		0									
	Circulation	0		0									
	Neurological Condition	0		0									
	Fluid Balance	0		0									
	Blood Balance	0		0									
	Hemofiltration	0		0									
	Pain Assessment	60		70									
	Pressure Sore Risk Assessment	20		15									
	Glasgow Coma Scale	10		10									
	Nurse Notes	50		80									
Total										3.934	7.343	3.409	46,43%
Total (min.)										66	122	56,8	46,43%
**Total (hours)**										**1,09**	**2,04**	**0,95**	**46,43%**

A different result from all other recorded time measurements draws attention to the patient identification form. As seen in Table 1, the digital and paper form filling time spent by Nurse X on the Patient Identification Form was equal to each other. In contrast, the filling time in the digital environment was shorter than the filling time in the paper environment in all form types. This can be interpreted that the more experienced nurse fills out paper forms more practically. Considering, however, the average time required to fill out forms in the digital environment is calculated to be 4 s (17.39%) faster per day. Accordingly, the daily repetition coefficient (R) was recognized as 1/30 since the Patient Identification Form, which is included in the Planning and Care Forms and filled in the first visit to the ICU, is repeated once a month.

Similarly, the forms included in the Planning and Care Forms (Diabetes Form, Pressure Sore Risk Assessment Form, and Nutrition Screening and Nutrition Risk Assessment Form) are filled out twice a day. In daily evaluation, the measured savings in time is 150 s (2.5 min, 83.3% of the total time) for Diabetes Forms, 135 s (2.25 min, 75% of the total time) for Pressure Sore Risk Assessment Forms, and 420 s (7 min, 87.5% of the total time) for Nutrition Screening, and Nutrition Risk Assessment Forms.

Given the Daily Follow-up Form, the difference between the time for filling out paper forms and the time for filling out digital forms was measured at an average of 2,700 s (45 min) per patient, and it was calculated that 41.67% of the time was saved when filling out paper forms.

Considering the nursing forms filled out daily in Table 1, it was measured that a total of 2.04 h per nurse/day are spent filling out forms in this paper environment. If the same forms are filled in the digital domain, this time is reduced to 1.09 h with 57 min saved. This demonstrates that digitalization holds 46.43% of the time for filling out ICU daily nursing forms.

### Effect of digitalization on paper consumption

The forms this study relies on may differ depending on the institutions, and the page numbers may vary. In our example, 5 daily forms consist of 18 pages in total.

When adding up the hospitalization days for 428 patients, they are found to care for 5,420 days. A total of 97,560 sheets of paper were used during the day of care. Given current toner costs, the calculated savings to be achieved per year is TRY 2,601.60 (US$ 464.5), resulting in the deliverance of TRY 4,487.76 (US$ 801.3) in annual paper consumption.

### National projection

If we make a rough projection regardless of the case complexities between hospitals and the difference between private and public hospitals;

Based on the data disclosed by the Ministry of Health in September 2020, Turkey has 28,353 adult intensive care beds and 68% occupancy rate [19]. According to this ratio, the number of occupied beds is 19,280. The study shows that 56.8 min are saved per occupied bed thanks to digitalization, corresponding to 3.95% of the daily care time. Accordingly, the number of nurses held per occupied bed in Turkey will be 760,71.

The gross salary of nurses in 2020 is about US$ 1,428.67 *(C: Monthly Gross Cost of Nurses).*

For 760,71 nurses, it is anticipated to achieve an annual cost savings of US$ 13,040,804.8 *(Nurse Cost Savings)*.

In Table 2, Turkey provided 7,037,214.60 days of care with a 68% occupancy rate in 28,353 beds. When the daily paper and toner consumption cost is multiplied by the days of care, it is predicted that the sum of US$ 1,654,323.36 will be saved per year.

**Table 2:** Total Paper and Toner Savings

Number of Intensive Care Beds in Turkey	Occuoancy Ratio	Days of Care	Total Paper Savings	Total Toner Savings	Total Paper &Toner Savings
28,353	0,68	7.037.214,60	$ 1.040.502,6	$ 603.189	**$ 1.643.692**

## Discussion

### Interpretation within the context of the broader literature

Digitalization has become a very significant issue in the health field, as in any other area. Studies are carried out on the storage and analysis of digitalized health data. The entire personal healthcare data and the data produced in clinical practices are combined and rapidly transferred to clinical practice, aiding personalized treatment decisions [20]. The COVID-19 outbreak has once again demonstrated the importance and contribution of digitalization [21]. While the studies conducted in this field are promising, the issues, such as the variety of data, how to store them, data sharing restrictions, privacy, and security, bring about many challenges [22]. Healthcare professionals are exposed to a high workload and time pressure. They are required to provide patient-centered care and respond to the needs of patient relatives. It is indispensable to focus on new solutions to spend time on patient care more effectively and mitigate the pressure on healthcare professionals [23]. Digitalization, new organizational structures, and business models are being developed in the healthcare workforce. Clinical informatics will play a very influential role in minimizing the losses in the force and achieving responsiveness [24].

Many studies are carried out for continuous improvement with the development of health practices in the world, transformation in organizational structures, and new business models. Therefore, it is striking that no similar study was conducted specifically on ICUs with the least digitalization in healthcare institutions. The study published by Asah in 2013 identified the reasons for nurses to use computers in the preparation of care plans (77%), access to information (55%), and monitoring of innovations (42%). Still, it failed to measure the effect of digitalization on time separately [25]. The study conducted in the ICU of Akdeniz University Hospital determined that nurses use technology effectively in patient care and adopt a positive attitude towards technology. Still, the concrete contributions of this attitude or its effect on quality were not measured [26]. Another study emphasized that the adoption of technology in intensive care supported the facilitation of nursing care processes; [27] however, the study failed to specify concrete indicators regarding the extent to which it facilitated these processes, as in other studies.

### Statement of principal findings

The literature contains no study in which the effect of digitalization on the nursing workforce and consumable costs (paper and toner) is measured in ICUs and projected on a national scale. One of the most recent studies analyzing the effect of digitalization on time and consumable costs in inpatient wards outside the ICU was conducted by Esra Volkan in a hospital in Turkey and published in 2019. If we compare both studies for having been born in the same country, our study reveals that digitalization reduced the time to fill out paper forms by 46.52% in the ICU. On the other hand, this rate turned out to be 65%, as demonstrated by the study conducted on other services. This difference may result from the conduct of hourly checks by nurses in ICUs to verify daily follow-up data, regardless of the use of digital forms.

### Strengths and limitations

Forms filled as standard and repeated at certain times were selected for all patients at the stages determined in the Intensive Care workflow. Forms used in exceptional cases (for some special cases) differ, so they are excluded.

While determining the limits of the study, the forms included in the standard procedure of the Ministry of Health and which must be applied for ICUs were chosen. In this way, it is aimed to minimize the differences that may arise between institutions.

### Implications for policy, practice, and Research

The cost of return on investment can be calculated by examining the difference between the acquisition costs of ICU information systems provided by other studies in this field and the savings achieved.

## Data Availability

The datasets used and analyzed during the current study are available from the corresponding author on reasonable request.
